# Survey of the *Bradysia odoriphaga* Transcriptome Using PacBio Single-Molecule Long-Read Sequencing

**DOI:** 10.3390/genes10060481

**Published:** 2019-06-25

**Authors:** Haoliang Chen, Lulu Lin, Minghui Xie, Yongzhi Zhong, Guangling Zhang, Weihua Su

**Affiliations:** Institute of Plant Protection and Agro-Products Safety, Anhui Academy of Agricultural Sciences, Hefei 230031, China; chenhaoliang@aaas.org.cn (H.C.); linlulu@aaas.org.cn (L.L.); xieminghui@aaas.org.cn (M.X.); zhongyouzhi@aaas.org.cn (Y.Z.); zhangguangling@aaas.org.cn (G.Z.)

**Keywords:** *Bradysia odoriphaga*, transcriptome, single-molecule long-read sequencing

## Abstract

The damage caused by *Bradysia odoriphaga* is the main factor threatening the production of vegetables in the Liliaceae family. However, few genetic studies of *B. odoriphaga* have been conducted because of a lack of genomic resources. Many long-read sequencing technologies have been developed in the last decade; therefore, in this study, the transcriptome including all development stages of *B. odoriphaga* was sequenced for the first time by Pacific single-molecule long-read sequencing. Here, 39,129 isoforms were generated, and 35,645 were found to have annotation results when checked against sequences available in different databases. Overall, 18,473 isoforms were distributed in 25 various Clusters of Orthologous Groups, and 11,880 isoforms were categorized into 60 functional groups that belonged to the three main Gene Ontology classifications. Moreover, 30,610 isoforms were assigned into 44 functional categories belonging to six main Kyoto Encyclopedia of Genes and Genomes functional categories. Coding DNA sequence (CDS) prediction showed that 36,419 out of 39,129 isoforms were predicted to have CDS, and 4319 simple sequence repeats were detected in total. Finally, 266 insecticide resistance and metabolism-related isoforms were identified as candidate genes for further investigation of insecticide resistance and metabolism in *B. odoriphaga*.

## 1. Introduction

*Bradysia odoriphaga* is a notorious pest that impacts the production of Liliaceae family vegetables, especially the Chinese chive (*Allium tuberosum*), which is one of the main ingredients in Chinese dumplings [1]. This pest can cause more than 50% loss of the Chinese chive production or even total destruction in the absence of a chemical control [2]. One reason for the difficulty in controlling *B. odoriphaga* is that most of its lifecycle is underground, and people only realize the damage when the chives stunt or even die. Another reason for difficulties controlling the pest is that its rapid life-cycle results in high resistance to commonly used insecticides [3,4]. As a result, pesticides are heavily applied to control this pest, which leads to environmental pollution and residual pesticide in crops [1,5].

With the development of next-generation sequencing technology, short- and long-read sequencing have increased dramatically in the past decade. Short-read sequencing is excellent for the production of high-quality deep coverage of the genomes. In *B. odoriphaga*, short-read sequencing was first used to sequence and characterize the larval transcriptome [6], after which the developmental stages transcriptomes were analyzed [7,8] and gene responses to different pesticides identified [9,10]. The odorant binding protein and chemosensory protein genes in *B. odoriphaga* were also identified through short-read sequencing [11]. However, the short-read length is limited in that complex regions with repetitive or heterozygous sequences may be misassembled [12], and PCR amplification bias during sequence library construction may cause the loss of a small portion of the sequence [13]. Long-read sequencing technologies can generally overcome those problems. When compared to other long-read sequencing platforms, the performance of the Pacific Biosciences (PacBio) sequence platform has the advantages of providing the longest read length, shortest time per run, and an acceptable price [14,15].

In this study, the PacBio platform was used to sequence the transcriptome of *B. odoriphaga* and produce a single-molecule long-read dataset. The isoforms after polishing and removal of redundancies were then annotated by software using the non-redundant nucleotide (NT), non-redundant protein (NR), Gene Ontology (GO), Cluster of Orthologous Groups (COG), Kyoto Encyclopedia of Genes and Genomes (KEGG), and SwissProt databases. Isoform functions were predicted by categorization of the isoforms by GO and COG, after which they were grouped into pathways using the KEGG. Simple sequence repeats (SSRs) in isoforms were identified, and the isoforms related to insecticide resistance and metabolisms were analyzed.

## 2. Materials and Methods 

### 2.1. Insect Rearing

Chinese chive maggots (*B. odoriphaga*) were collected in March 2016 from a farm in Bozhou, Anhui Province, China (115.89° E, 33.99° N). The maggots were reared on the stems of Chinese chives in Petri dishes in a walk-in climate chamber maintained at 25 ± 1 °C under a photoperiod of 14 h light: 10 h darkness and 70 ± 10% relative humidity [16]. Petri dishes were prepared by coating the bottom with 1.5% agarose gel and then placing filter paper on the gel after it solidified to keep it moist. After adult emergence in the Petri dish, several pairs of adults were introduced into plastic bowls containing 3–4 pieces of 1–2 cm chive stems using an aspirator, after which the bowl was covered with a lid. The adults then laid eggs on the pieces of chive stems, which were transferred to Petri dishes containing the gel and filter paper. Newly hatched larvae were subsequently transferred to new Petri dishes containing gel, filter paper, and chive stems at 7–10 days after the eggs were laid. The larvae were transferred to a new Petri dish containing gel, filter paper, and chive stems every 3–4 days until they pupated. Insects in different development stages including eggs, second instar larvae, fourth instar larvae, pupae, and adults were collected for RNA extraction.

### 2.2. RNA Isolation, cDNA Library Construction, and PacBio ISO-Seq

SV Total RNA Isolation System (Promega Corporation, Madison, WI, USA) was used to isolate the *B. odoriphaga* eggs, second instar larvae, fourth instar larvae, pupae, and adult total RNA according to the manufacturer’s protocols, after which possible residual genomic DNA was removed with deoxyribonuclease (DNase I: Fermentas Inc., Burlington, ON, Canada). The RNA integrity was determined using Agilent RNA 6000 Nano Reagents Port 1 (Santa Clara, CA, USA) on a Agilent 2100 Bioanalyzer (Santa Clara, CA, USA) (Table S1). Total RNA from different developmental stages was mixed as one sample for cDNA library construction. cDNA synthesis and library construction was conducted using a Clontech SMARTer PCR cDNA Synthesis Kit (catalogue number: 634925) (Clontech, Mountain View, CA, USA). Sequencing was conducted on the Pacific Biosciences RS II platform (Pacific Biosciences, Menlo Park, CA, USA). BluePippin (Sage Science, Beverly, MA, USA) was used for selection of cDNA sequences ranging in size from 1 to 2 kb, 2 to 3 kb, and 3 to 6 kb. Three, three, and two SMRT (Single-molecule real-time sequencing) cells were used to sequence the 1–2 kb, 2–3 kb, and 3–6 kb libraries, respectively, and the reads were deposited in the NCBI (National Center for Biotechnology Information) Sequence Read Archive database under the accession numbers SRR8903502, SRR8903501, and SRR8903503.

### 2.3. PacBio ISO-Seq Data Polish

The standard bioinformatics analysis pipelines to obtain high-quality consensus full-length isoforms include reads of inserts (ROIs), classification, cluster analysis, and removal of redundancy. ROIs were used to process reads from the insert sequence of individual molecules and to estimate the length of the insert sequence loaded into an SMRT cell. Classification was used to classify ROIs into full-length or non-full-length transcripts detected by 5’ and 3’ primers and possibly the poly A tail. The reads of the inserts containing the 5’ primer, 3’ primer, and poly A tail were defined as full-length transcripts; other reads of the inserts were defined as non-full-length transcripts. Clustering was used to predict consensus isoforms from classified full-length transcripts using the Interactive Clustering and Error Correction (ICE) algorithm first, after which Quiver v1.1.0 (Pacific Biosciences of California Inc., Menlo Park, CA, USA) [17] was run to polish the consensus isoforms using non-full-length transcripts. The polished isoforms were defined as high-quality or low-quality isoforms based on the Quiver accuracy. The minimum Quiver accuracy needed to classify an isoform as high quality was 0.99 for libraries below 3kb, while for libraries of 3–6 kb, it was 0.98. Finally, the high-quality isoforms were merged and redundancy was removed to obtain the final consensus isoforms.

### 2.4. Isoforms Functional Annotation

Polished isoforms were subjected to BLAST (v2.2.23) (NCBI, Bethesda, MD, USA) [18] searches of the NR, COG, KEGG, and Swiss-Prot databases (with significant thresholds of E-value ≤ 1 × 10^−5^). After isoforms were NR annotated, Blast2GO (v2.5.0) was used to analyze GO term annotations [19], and the isoforms were finally assigned into three ontologies: molecular function, cellular component, and biological process [20]. InterProScan5 (v5.11) (European Molecular Biology Laboratory, Cambridgeshire, UK.) [21] was used to scan for the presence of domains and important sites in isoforms and obtain the InterPro annotation.

### 2.5. Coding DNA Sequence (CDS) Prediction and Simple Sequence Repeat (SSR) Detection

Using functional annotation, we selected the segment of the transcript that best mapped to functional databases in a priority order of NR, SwissProt, KEGG, and COG as its CDS. Transcripts that could not be aligned to any database mentioned above were predicted using ESTScan (v3.0.2) (Swiss Institute of Bioinformatics, Lausanne, Switzerland) [22] with BLAST-predicted CDS as a model. MISA (v1.0) (Leibniz Institute of Plant Genetics and Crop Plant Research (IPK) Gatersleben, Seeland, Germany.) [23] was used to find SSR in isoforms. Di-nucleotide repeats of more than six times, tri-nucleotide and tetra-nucleotide repeats of more than five times, and penta-nucleotide and hexa-nucleotide repeats of more than four times were considered to be SSRs.

## 3. Results and Discussion

### 3.1. Sequencing and Data Polishing

**Figure 1:**
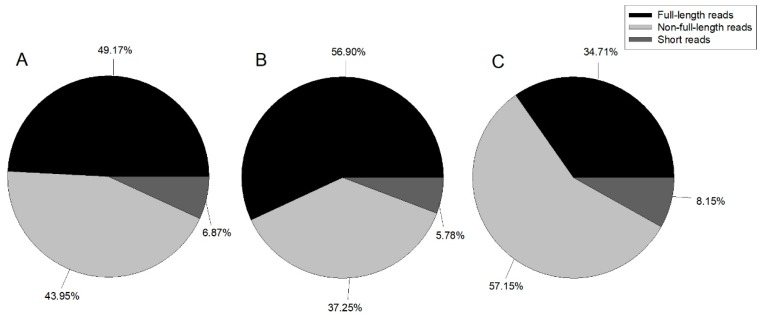
Pie chart of ROI (reads of inserts) classification summary. A: 1–2 kb, B: 2–3 kb, and C: 3–6 kb.

In this study, we constructed three ISO-Seq libraries for one sample and sequenced eight cells in total using the Pacific Bioscience RS II platform. Finally, 489,270 ROIs with 1,041,604,967 bp were generated (Table 1). Reads were classified by whether the 5’ primer, 3’ primer, or poly A tail were detected, with reads containing 5’ primer, 3’ primer, and poly A tail considered as full-length; those with the 5’ primer and 3’ primer, 3’ primer and poly A tail, or 5’ primer and poly A tail classified as non-full-length; and all other reads classified as short reads. Figure 1 illustrates the proportion of each category.

Although PacBio single molecule sequencing yields long reads, it has a high error rate; therefore, we used Quiver to polish the consensus isoforms. The basic steps were as follows: full-length reads were clustered into consensus reads. For each cluster, if there was sufficient full-length and non-full-length coverage, then Quiver was run to polish the consensus. Depending on the Quiver output quality value, which indicated how confident the consensus calls were, the script binned the Quiver polished output as either high or low quality. Cluster summaries of each library are shown in Table 2. High-quality consensus sequences were subjected to further analysis. High-quality consensus isoforms of each library were merged into the final result, and redundancy was removed. The isoforms are summarized in Table 3. A total of 39,129 isoforms were obtained with a total length of 76,617,709 bp, a mean length of 1958 bp, and an N50 length of 2044 bp (half of the isoforms is larger than or equal the 2044 bp). This number of isoforms is lower than that reported by Gao et al. [7], who found a transcriptome of 47,578 unigenes, but higher than that reported by Chen et al. [6], who assembled 16,829 unigenes. This may have been because Gao et al. [7] used three developmental stages (third-instar, fourth-instar, and pupa) for de novo transcriptome assembly, but Chen et al. [6] only used third-instar larvae for unigene assembly, and some genes were only expressed in the specific development stage. The number of unigenes generated from the three developmental stages in Gao’s study was greater than the number of isoforms generated from all development stages in our study. This may have been because some unigenes only have the partial sequence of the genes, and the isoforms generated in this study could produce the full length of the genes. This also explains why the mean length and N50 in this study (1958 bp and 2044 bp, respectively) are much longer than those reported by Gao et al. [7] and Chen et al. [6], who reported mean lengths of 860 bp and 1517 bp and N50 lengths of 576 and 762 bp, respectively.

### 3.2. Functional Annotation

**Figure 2:**
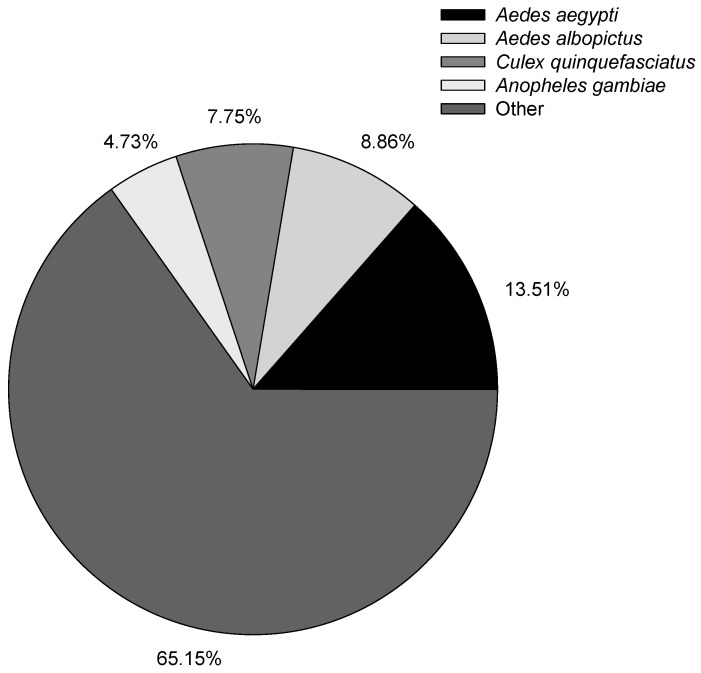
Distribution of annotated species.

After obtaining the isoforms, we used BLAST, Blast2GO, and InterProScan5 to perform functional annotation based on the NR, NT, GO, COG, KEGG, Swiss-Prot, and InterPro databases (Table 4). Overall, 91.10% of the isoforms had annotation results with different databases. In the previous *B. odoriphaga* short-read sequence transcriptome study, 12,480 (74.16%) out of 16,829 and 21,985 (46.21%) out of 47,578 unigenes had annotation results against different databases in Chen’s and Gao’s studies, respectively [6,7]. The higher annotated percentage of the isoform shows the good quality of long-read sequence results obtained using the Pacific Bioscience RS II platform. The species distribution with NR annotation is shown in Figure 2, and the most annotated species were mosquitoes, with 13.51% of the isoforms closely matching *Aedes aegypti*, followed by *Aedes albopictus* (8.86%), *Culex quinquefasciatus* (7.75%), and *Anopheles gambiae* (4.73%). Evaluation of the species distribution confirmed that *B. odoriphaga* belonged to the suborder of Nematocera rather than Brachycera. The larvae of *B. odoriphaga* look like fly larvae, but the adults look like mosquitos, which has led to confusion regarding which sub-order they belong to. However, the species distribution showed that the top four species were midges, indicating that *B. odoriphaga* are more closely related to midges than flies.

**Figure 3:**
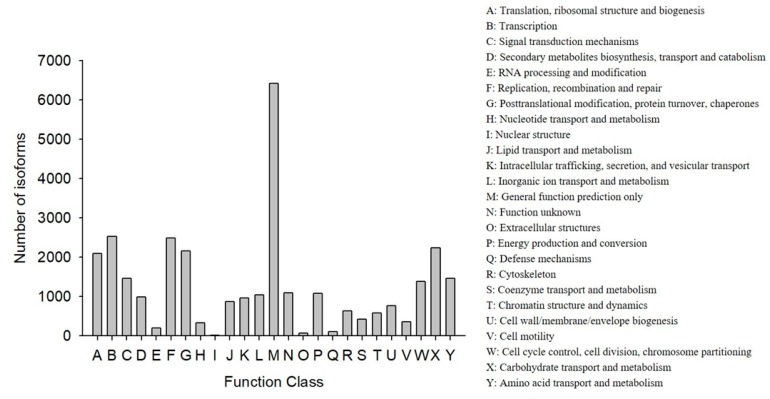
Functional distribution of the Cluster of Orthologous Groups (COG) annotation. The x-axis represents the COG function category. The y-axis represents the number of isoforms.

COG classification showed that 18,473 out of the 33,238 NR hit isoforms could be categorized (Table 4). Among the 25 COG categories, the largest group was the cluster for “general function prediction only” (6417, or 20.27%), followed by “transcription” (2523, or 7.97%), and then “replication, recombination, and repair” (2483, or 7.84%), while the categories of “nuclear structure” (16, or 0.05%), “extracellular structures” (65, or 0.21%), and “defense mechanisms” (110, or 0.35%) were the smallest clusters (Figure 3).

**Figure 4:**
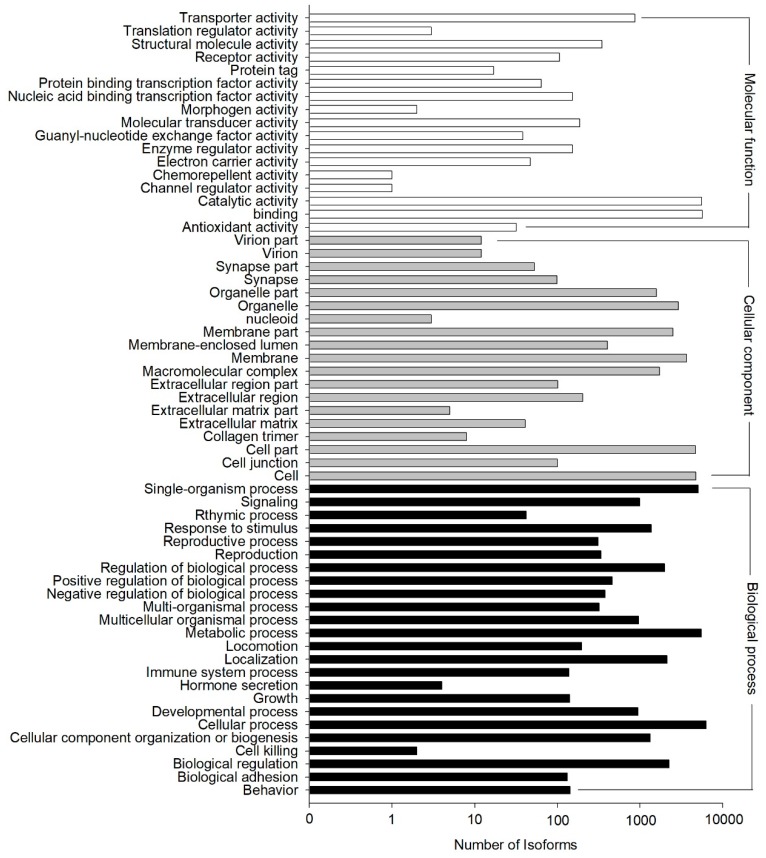
Functional distribution of Gene Ontology (GO) annotation. X-axis represents the number of isoforms. Y-axis represents the Gene Ontology function category.

The GO annotation results showed that 11,880 out of the 33,238 NR hit isoforms could be assigned to 67,635 GO terms, with the three main categories of biological process, cellular component, and molecular function assigned 13,312, 22,878, and 31,445 terms, respectively (Figure 4). GO terms were finally assigned into 60 functional groups, and the number of “cellular process” terms (6314 terms) was the largest for “biological process”, “cell” (4742 terms) for “cellular component”, and “binding” (5704 terms) for “molecular function.” In contrast, there were only two terms in the cluster of “cell killing”, which was among the main categories of “biological process”, “nucleoid” (three terms), the main category of “cellular component”, and both “channel regulator activity” (one term) and “chemorepellent activity” (one term) from the category of “molecular function”. (Figure 4).

**Figure 5:**
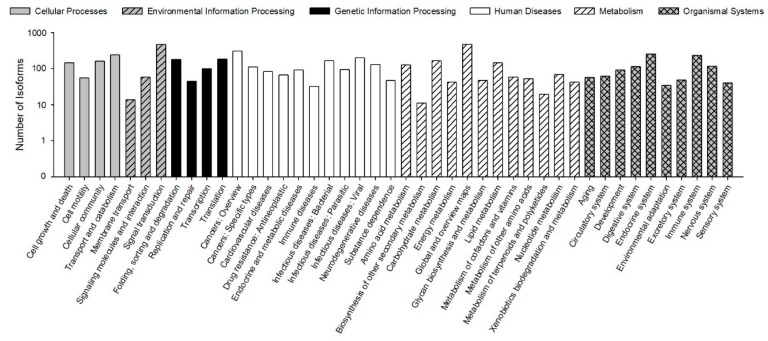
Functional distribution of Kyoto Encyclopedia of Genes and Genomes (KEGG) annotation. The x-axis represents the KEGG function category. The y-axis represents the number of isoforms.

The KEGG pathways results showed that 30,610 (78.23% of 39,129) isoforms were mapped into different KEGG pathways. The pathways most assigned by the isoforms were “signal transduction” (4720, 8.92%), followed by “global and overview maps” (4672, 8.83%), and “cancers: overview” (3072, 5.80%) (Figure 5). The KEGG pathway could provide basic information for isoforms involved in the specific processes and pathways during *B. odoriphaga* research. For example, 428 isoforms were mapped into “xenobiotics biodegradation and metabolism,” and this pathway is closely related to insecticide detoxification and metabolism.

### 3.3. CDS Prediction and SSR Detection

**Figure 6:**
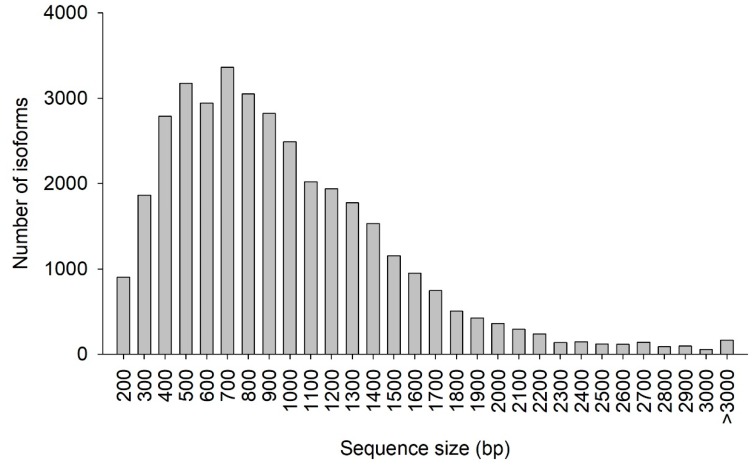
Coding DNA sequence (CDS) length distribution. The x-axis represents the length of CDS. The y-axis represents the number of CDS.

The CDS prediction summary is shown in Table 5. Overall, 93.07% of the isoforms were predicted to have CDS. Additionally, the predicted CDS had a mean length of 911 bp and N50 of 1128 bp. The distribution of the CDS length and number of isoforms are shown in Figure 6. The greatest abundance of CDS was between 400 and 1000 bp, where 56.65% of all CDS were located. In total, 4319 SSRs were detected, with tri-nucleotide repeat motifs the most common (2565, 59.34%), followed by di-nucleotide (1569, 36.33%), tetra-nucleotide (99, 2.29%), penta-nucleotide (67, 1.55%), and hexa-nucleotide (19, 0.44%) repeat motifs (Table 6).

### 3.4. Insecticide Resistance and Metabolism-Related Isoforms

**Figure 7:**
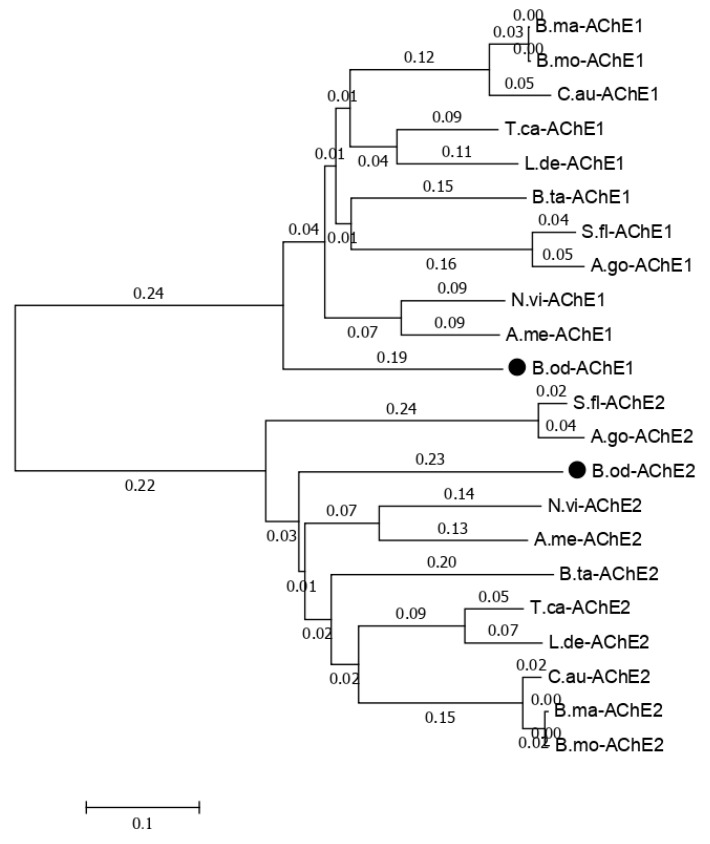
Phylogenetic relationships of the two *Bradysia odoriphaga* acetylcholinesterase genes (AChEs) with those from other insects. A phylogenetic tree was generated using a Clustal W alignment and neighbor-joining method with 1000 bootstrap replicates in MEGA 7.0 (Molecular Evolutionary Genetics Analysis Version 7.0). The GenBank (a comprehensive database that contains publicly available nucleotide sequences) accession numbers of each AChE are listed as follows. *B. odoriphaga*, B.od-AChE1 and B.od-AChE2; *Tribolium castaneum*, T.ca-AChE1 (NP_001280548.1) and T.ca-AChE2 (NP_001280548.1); *Sipha flava*, S.fl-AChE1 (XP_025418725.1) and S.fl-AChE2 (XP_025418373.1); *Nasonia vitripennis*, N.vi-AChE1 (XP_008214577.1) and N.vi-AChE2 (XP_001605568.2); *Leptinotarsa decemlineata*, L.de-AChE1 (XP_023011979.1) and L.de-AChE2 (XP_023021546.1); *Chilo auricilius*, C.au-AChE1 (KF574430.1) and C.au-AChE2 (KF574431.1); *Bombyx mandarina*, B.ma-AChE1 (XP_028039466.1) and B.ma-AChE2 (XP_028031078.1); *Bemisia tabaci*, B.ta-AChE1 (XP_018895731.1) and B.ta-AChE2 (XP_018898239.1); *A. mellifera*, A.me-AChE1 (XP_016770282.2) and A.me-AChE2 (NP_001035320.1); *Aphis gossypii*, A.go-AChE1 (XP_027848418.1) and A.go-AChE2 (XP_027850887.1); and *Bombyx mori*, B.mo-AChE1 (XP_012552768.1) and B.mo-AChE2 (NP_001108113.1).

The functions annotated in the NR database related to cytochrome P450, glutathione transferase, carboxylesterase, trypsin, NADH dehydrogenase, catalase, sodium channel, acetylcholinesterase, superoxide dismutase, nicotinic acetylcholine receptor, and GABA receptor in this study and previous studies [6] are listed in Table 7. Less insecticide resistance and metabolism-related genes were generated in this study than in the results of the larval transcriptome (Table 7), which may have been because the isoforms generated in this study could contain the full length of the genes, but those observed in previous studies only contained the partial sequence of the genes. There were 107 isoforms related to Cytochrome P450, which is a reasonable number for insect species because the number of P450 genes in insects ranges from 45 in *Apis mellifera* to 204 in *C. quinquefasciatus*, and there is an average of around 100 P450 genes in insect species [24,25,26]. Usually, insects have one or two acetylcholinesterase genes, but based on previous *B. odoriphaga* transcriptome data [6], there are eight unigenes related to the acetylcholinesterase gene. In this study, we identified two isoforms of the acetylcholinesterase gene (Figure S1). The phylogenic tree for the acetylcholinesterase-related isoforms with other insects that have two acetylcholinesterase genes shows that *B. odoriphaga* contains two acetylcholinesterase genes (Figure 7).

## 4. Conclusions

In this study, the transcriptome including all developmental stages of *B. odoriphaga* was first sequenced by Pacific single-molecule long-read sequencing. Finally, 39,129 isoforms were generated, 35,645 of which had annotation results when checked against different databases. CDS prediction showed that 36,419 out of 39,129 isoforms were predicted to have CDS, and 4319 SSRs were detected in total. Finally, 266 insecticide resistance and metabolism-related isoforms were identified, and those genes could be considered candidate genes for further investigation of insecticide resistance and metabolism in *B. odoriphaga*.

## Figures and Tables

**Table 1:** Summary of reads information for libraries of each size.

Library	Cell Number	Reads Number	Read Bases (bp)	Mean Read Length (bp)
1–2 kb	3	170,064	300,226,655	1765
2–3 kb	3	214,887	440,424,904	2049
3–6 kb	2	104,319	300,953,408	2884

**Table 2:** Cluster summary for each library.

Library	Cluster Type	Total Isoforms	Total Base (bp)	Mean Quality	Mean Isoform Length (bp)
1–2 kb	High quality	18,075	25,545,296	0.9971	1413
1–2 kb	Low quality	6003	10,847,589	0.3498	1807
2–3 kb	High quality	24,983	48,797,309	0.9972	1953
2–3 kb	Low quality	6994	15,962,260	0.5709	2282
3–6 kb	High quality	10,631	29,644,232	0.9952	2788
3–6 kb	Low quality	4414	13,685,764	0.4393	3101

**Table 3:** Number of isoforms.

Library	Library Isoforms	*Bradysiaodoriphaga* Isoforms
1–2 kb	18,075	16,226
2–3 kb	24,983	21,241
3–6 kb	10,631	9140
Total	53,689	39,129

**Table 4:** Summary of functional annotation results.

Values	Total	NR	NT	Swiss-Prot	KEGG	COG	InterPro	GO	Overall
Number	39,129	33,238	20,662	31,343	30,610	18,473	29,951	11,880	35,645
Percentage (%)	100	84.94	52.80	80.10	78.23	47.21	76.54	30.36	91.10

Overall: the number of isoforms annotated with at least one functional database. NR: Non-Redundant protein, NT: Non-redundant nucleotide, KEGG: Kyoto Encyclopedia of Genes and Genomes, COG: Cluster of Orthologous Groups, GO: Gene Ontology.

**Table 5:** Quality metrics of predicted coding DNA sequence (CDS).

Software	Total Number	Total Length (bp)	Mean Length (bp)	N50 (bp)	GC (%)
BLAST	34,782	31,090,851	893	1104	43.61
ESTScan	1637	2,111,526	1289	1446	40.73
Overall	36,419	33,202,377	911	1128	43.42

N50: 50% of the total length is contained in CDS greater than or equal to this value. GC (%): the percentage of G and C bases in all CDS. BLAST: Basic Local Alignment Search Tool, ESTScan: Expressed Sequence Tag Scan.

**Table 6:** Distribution of Single Sequence Repeats (SSRs) based on the number of repeat motifs.

Nucleotides of Motif	Di-	Tri-	Tetra-	Penta-	Hexa-	Total
Repeats						
4	-	-	-	61	18	79
5	-	1782	74	5	1	1862
6	643	506	12	1	0	1162
7	322	158	3	0	0	483
8	209	64	3	0	0	276
9	132	37	1	0	0	170
10	93	3	0	0	0	96
11	59	7	0	0	0	66
12	38	3	1	0	0	42
13	15	3	1	0	0	19
14	14	1	1	0	0	16
15	13	1	1	0	0	15
16	6	0	0	0	0	6
17	6	0	2	0	0	8
>18	19	0	0	0	0	19
Total	1569	2565	99	67	19	4319

**Table 7:** Isoforms related to insecticide resistance and metabolism.

Gene Name	Number of Isoforms with Hits in NR Database	Number of Unigenes with Hits in NR Database [6]
Cytochrome P450	107	158
Glutathione S-transferase	28	64
Carboxylesterase	22	37
Trypsin	61	48
NADH dehydrogenase	14	66
Catalase	11	3
Sodium channel	9	9
Acetylcholinesterase	2	8
Superoxide dismutase	5	9
Nicotinic acetylcholine receptor	3	5
GABA receptor	3	1

Number of isoforms or unigenes indicates BLAST hit with corresponding proteins in the NCBI NR (National Center for Biotechnology Information Non-Redundant protein) database (E-value ≤ 1 × 10^−5^).

## Supplementary Materials

The following are available online at https://www.mdpi.com/2073-4425/10/6/481/s1; Table S1: Values of samples detected by Agilent 2100, Figure S1: Sequences of AChE1 and AChE2 in *B. odoriphaga*.
