# Supplementary material for: Survey of the Bradysia odoriphaga Transcriptome Using PacBio Single-Molecule Long-Read Sequencing
Source: Genes (Basel). 2019 Jun 25;10(6):481. doi: 10.3390/genes10060481 (PMC6627194; doi:10.3390/genes10060481)
Supplement: Supplementary file 1 [file genes-10-00481-s001.zip › Figure S1.pdf]

Figure S1: Sequences of AChE1 and AChE2 in *B. odoriphaga*

B.od-AChE1

>[Bradysia_odoriphaga]_6535 2770bp

AATCTCAGTTCAGATAGTGGTACCAAACTGCGGTGTTCGAGAGAAACTAATAAGTGAA
TACAAAAAAAAAGGTTTTTTTTGTGGAATATAACAATTATTAAACTTTAGTGGTGACATT
TGAGTTAAAAATCACATTGAAGTAGACAGTAGAGAAATATAATTTCTAAATGAAAAATT
AAACAATAAATTCAAACTGAAAATTGTTTTTTTAAGGAAAAATTACAATTCGAATAAAC
AGCCAGTGTTCAAGTGTATGTGTATGTGTGTTTAGGACAAATTTTCCGATCCAAAAATT
ATACTAAAGACAAAATCGGTGGATGATATGAAAAATTATAAATATACGATTAACTAACTA
TCCGGGGACTTTGCTGATTTATTTTGCTGATTACATAGGAGAGCTTTTTGTTTGTTTGTT
TGTTTTGTTTTTAAGGAGAGACGATAGAAGACAATCGAGCCGCCAAAACCAACCGGAC
ACCCGCTAAGTAATATGGTGCAAGTGATGAACTTCCGGTGTTGACATAAAAATTCTCCA
AAATGGAGAAACACAGTGGACATTATCGATTCGGTTTAATGCCGCGGCCTATACGACTA
CGCCAATTATTATTATGTACAATAGGAATGCTCGGTGTATTCGACCATATACTGGTCGAAT
GCCGGCATCGTGAATTAGAGAATGCTCAAATTCAATTAAGTCAAAAACACAATTCACA
ATCATCGTCGGTGCCAAATGATGATGATGCCTTTTTTACCCCATATCTTGGTCATGGTGA
AGCCGTCAGAGTATTAGATGCAGAGCTGGGAACTTTGGAAATGGAACAATTTAGGGAT
ACCAAAGCGTTAACACGCCGGAGAGGCGTCAGTAGAAGGGAATCATCGTCGCATGATC
CAGACGACGACGATCCGATGGTAATCACAACCGACAAAGGTAAAATTCGAGGCACAG
TGCTGACATCGGGTAGTGGACGAAAGGTCGATGCATGGCTCGGCATACCATATGCGCA
ACCACCAATCGGTGCATTACGCTATCGACTTCCACGGCCTATCGAACGATGGACTGGCG
TTCGAAACGCAACAACACCATCGAATTCCTGTGTTCAAATTGTGGATGTTGTATTTGGC
GATTTTCCGGGCGCCACAATGTGGAATCCCAATACGCCACTATCCGAAGATTGTCTCTA
CATAAATGTGGTGGTTCCTAGGCCTAGACCAAAAATGCGCCTGTAATGTTGTGGATATT
TGGAGGTGGATTTTATTCGGGCACTGCAACGCTAGATGTGTACGATCACAAAACACTTG
CTGCTGAAGAGAATGTCATTGTCGTTTCAATGCAATACAGAGTGGCGAGTCTTGGTTTC
CTTTACCTGGGAACACCAGATGCACCAGGAAATGCTGGTCTCTTTGATCAAAATTTAGC
ACTGAGATGGGTACGAGACAACATCCATAAATTTGGTGGCGATCCCAACCGAGTCACT
TTGTTCGGCGAAAGTGCCGGCGCCGTATCGGTGTCGATGCACTTACTTTCATCCCTCTC
ACGAGATCTATTTCAACGAGCCATATTAGAGAGTGGCTCTCCAACAGCTCCCTGGGCTT
TAATATCCAGAGAAGAAGCTATTTTAAGAGCTCTACGTTTAGCAGAAGCTGTCGATTGT
CCACATGATCGTCACAAGCTAACCGACACCGTTGAATGTCTACGTACGAAGGACGCTA
AAGAATTGGTTGATAACGAATGGGGAACGTTAGGAATATGCGAGTTTCCCTTCGTACCG
GTTGTGGATGGTGCATTTTTGGATGAAACACCACAAAAAAGTCTTGCCCACGGCCGAT
TCAAAAAGACAGAAATCTTAACCGGCAGCAACACCGAAGAGGGTTACTATTTTATAAT
TTATTATCTAACGGAACTGTTACGCAAAGAGGAGGGCGTGACGGTGTCTCGTGAAGAA
TTTTACCAGGCTGTGAAAGAACTTAATCCATATGTGAATAATCCAGCTAGACAGGCTATT
ATCTTCGAATACACTGATTGGATCGAACCCGATAATGTACACAACAATCGAGATGCACT
TGATAAAATGGTGGGTGACCTGCATTTCACATGCAATGTTAACGAATTTGCTAATAGATA
TGCCAAAGAAGGACTTAACGTTTACATGTATTTGTATACACATCGAACGAAAAGTAATC
CCTGGCCTCGATGGACTGGCGTTATGCATGGCGATGAAATTAATTACATTTTCGGTGAG
CCATTAAATCCGAATCACAGTTACACTGAGGAGGAAAAGGACTTTAGTCGCAAAATTA
TGAGATACTGGAGTAATTTTGCTAAGTACGGAGATCCGAATGGTGCGACACCAGATGTT
CAAGTATGGCCTAAACATACTGCAACTGGAAAACATTACTTGGAGTTGGGTATAAATAC

GACTCATGTGGGACGAGGACCACGGCTTAGACAATGTGCATTTTGGAAAGAGTATTTA
CCCCAATTGATACAAGCGACATCGCAAACATCAGCTAATCCTACAAGTGAACCATGTGC
AAATAGTGGCGCTTTAAATGGACACAGTCTCCTATTTCTAATAATAAGTTTTTTCATTTG
CATTTTACGTTTCGAAAATTTAATTCAAATTTAAAGCGAATTTAAATAAATTGTTTTCTGT
TCATTTGATTAAAATTTTTAACATAAAAATATATTTATAGAAACAAGAAAACAAACAGA
AAATTTGATATAGAGAGGGATGTTTCAACTATACATTGGCTATGCTTTATTAAAGTAAAC

Translated amino acid sequence
MLWIFGGGFYSGTATLDVYDHKTLAAEENVIVVSMQYRVASLGFLYLGTPDAPGNAGLFD
QNLALRWVRDNIHKFGGDPNRVTLFGESAGAVSVSMHLLSSLSRDLFQRAILESGSPTAPW
ALISREEAILRALRLAEAVDCPHDRHKLTDTVECLRTKDAKELVDNEWGTLGICEFPFVPV
VDGAFLDETPQKSLAHGRFKKTEILTGSNTEEGYYFIIYYLTELLRKEEGVTVSREEFYQAV
KELNPYVNNPARQAIIFEYTDWIEPDNVHNNRDALDKMVGDLHFTCNVNEFANRYAKEG
LNVYMYLYTHRTKSNPWPRWTGVMHGDEINYIFGEPLNPNHSYTEEEKDFSRKIMRYWS
NFAKYGDPNGATPDVQVWPKHTATGKHYLELGINTTHVGRGPRLRQCAFWKEYLPQLIQ
ATSQTSANPTSEPCANSGALNGHSLLFLIISFFICILRFENLIQI

B.od-AChE2
>[Bradysia_odoriphaga]_9736 2316bp
AGTTTTATTTGAGCCGTTGATTGTTACAGACAGAACATTTTGAACGTATTCGTTTAACCG
CATATATTATACACATCCCATAAATACAACCAACAACAAAAAAGTTTAATAAAAAGAAA
AATTTACCAATCATAAAGTGTTCGAATTAAAATACCAAAACACAGAAAAAAACGACAG
ACAACAAACTTAAAATTATAATGTAGTCAGTAAAAAAATGATAAAAACAAATCATTTAA
AAAAATAGTTCAACGTATCACTCGACAGTAACACGCGAGGTTTTTAAAGTAGAAAGTG
GAGAAAAATAGTAAAATAGAAAGAAAGGAAAGGAGAAAAAATATTTTCTATTTTTATT
CAACCACCTCAATTATGTTCACGCAATTAGTGCCGCTCGTTAGATTACTTATATGTGCCC
AAGCTATAACATCTGTGTTCGGGATAATCGATCGTCTGGTGGTGCAAAACCAGTTCAGG
TCCCATCCGCGGTCGTTCGGTCAAAGTTGAGGGTCGTGAAGTGCATGTGTATACCGGC
GTTCCGTTTGCAAAGCCACCCATCGACGCATTACGTTTCCGTAAGCCACTTCCAGCCGA
ACCGTGGCATGGTGTTCTGGATGCTACACGTTTGCCGCCCACATGCGTTCAAGAACGG
TATGAATACTTTCCCGGATTTCAAGGAGAGGATATGTGGAACCCCAACACAAATACATC
GGAAGATTGTCTGTATTTAAATGTTTGGGCACCGGTGAAACCGAAATTGCGACATGGA
CGCGGTGCTAATGGAGGCATTGAACATTCAGCCGATTCCGAACATATGAGCGATGAGG
AGAGCCCATCGAAAACTGGCCTTCCAATGTTAATTTGGCTATATGGCGGTGGTTTCATG
AGTGGTTCGTCAACATTAGATATTTATAATGCGGAAATATTGGCTGCGGTTGGAAACGT
GATTGTTGCATCGATGCAGTATCGTGTTGGTGCATTTGGATTTTTGTATCTGTCACCAAT
GTTGCCGGGAAATGAGGACGAAGCGCCAGGTAACGTTGGTATGTGGGATCAAGCATTG
GCTATTCGATGGTTGAAAGATAATGCACGGGGATTCGGTGGTGATCCGGACCAAATTAC
ATTATTTGGCGAATCAGCTGGTGGTAGTGCGGTTAATCTACATTTACTGTCACCTGTGAC
AAGAGGCCTAGCTAGACGTGGTATTTTACAATCAGGTACGTTGAATGCGCCATGGTCGC
ATATGTCGGGTGAACGTGCTGTCGAAATTGGTATGTCATTGATTGACGATTGTAACTGTA
ATTCATCGATGTTGAGGGAATCTCCAGGGGTTGTGTTGGCGTGTATGCGTAGCGTCGAT
GCGAAAACAATTTCGGTGCAACAATGGAACTCTTACTCGGGGATTCTAGGCTTTCCATC
AGCTCCAACCGTCGATGGTGATTTCATGCCAGCGGATCCAATGACAATGTTGGCAGAG

GCGGACTTGAGTGAAATTGACATTCTCGTTGGAAGCAATAAGGATGAAGGCACCTACT
TCCTGCTGTACGACTTTATTGACTACTTTGAAAAAGATTCACCGACGGCGTTACCGCGA
GAGAAATTCCTTGAAATAATGAACACAATATTCAGTAAAGCATCGGAGGCTGAACGTG
AAACCATAATATTTCAGTACACGAATTGGGAGAGCGTTGCTGATGGTTACCAGAATCAA
TATCAGGTTGGTCAAGCGGTTGGTGATCACTTTTTCATATGTCCAACAAACGAATACGC
TCAAGGAATGACTGAACGAGGAGCATCAGTAAAATATTACTATTTCACTCATCGAACCA
GTACATCGTTATGGGGTGAGTGGATGGGTGTCTTACACGCTGATGAAATTGAGTACATA
TTTGGCCAACCGATCAATAAATCACTACAGTATCGAGAACGCGAACGAGAATTAAGCC
GTAGAATGGTGCATGCAGTTAGTGAATTCGCTAGAACCGGCAATCCGGCACCTGATGG
CGAGACATGGCCTGAATATTCGAAAGAGAATCCCGTTTATTATATTTTTAACGCTGAAG
GTTCGAAAGGTGATGAAAAGCAGAAAGCTGACAAACTGGGAAAAGGACCGATGGCA
ACTGCCTGTGCATTTTGGAATGACTATCTACCGCGACTACGAATGTGGGCAGAGCCCCA
GAATTTGCCATGCATGAATTAGAAGATAGCAGCAGAACTTCGACTTCAACACATATATC
CGCCCCATCTATGAGTATTGCAATGCTAGTTCTATTGCTAGCTACACACCTATTAACAGG
GATGTAAACACATATCAATTTACac

Translated amino acid sequence
MWNPNTNTSEDCLYLNVWAPVKPKLRHGRGANGGIEHSADSEHMSDEESPSKTGLPMLI
WLYGGGFMSGSSTLDIYNAEILAAVGNVIVASMQYRVGAFGFLYLSPMLPGNEDEAPGNV
GMWDQALAIRWLKDNARGFGGDPDQITLFGESAGGSAVNLHLLSPVTRGLARRGILQSG
TLNAPWSHMSGERAVEIGMSLIDDCNCNSSMLRESPGVVLACMRSVDAKTISVQQWNSY
SGILGFPSAPTVDGDFMPADPMTMLAEADLSEIDILVGSNKDEGTYFLLYDFIDYFEKDSPT
ALPREKFLEIMNTIFSKASEAERETIIFQYTNWESVADGYQNQYQVGQAVGDHFFICPTNE
YAQGMTERGASVKYYYFTHRTSTSLWGEWMGVLHADEIEYIFGQPINKSLQYRERERELS
RRMVHAVSEFARTGNPAPDGETWPEYSKENPVYYIFNAEGSKGDEKQKADKLGKGPMAT
ACAFWNDYLPRLRMWAEPQNLPCMN
