# Supplementary material for: Survey of the Bradysia odoriphaga Transcriptome Using PacBio Single-Molecule Long-Read Sequencing
Source: Genes (Basel). 2019 Jun 25;10(6):481. doi: 10.3390/genes10060481 (PMC6627194; doi:10.3390/genes10060481)
Supplement: Supplementary file 1 [file genes-10-00481-s001.zip › Table S1.pdf]

Table S1: Values of samples detected by Agilent 2100

Development stages	Concentration (ng/μL)	Volume(μL)	total amount (μg)	OD260/280	RIN
Egg	804	60	48.24	2.14	7.1
Second instar larva	660	120	79.20	2.17	7.1
Fourth instar larva	800	140	112.00	2.16	6.9
Pupa	884	120	106.08	2.05	7.1
Adults	995	120	119.40	2.19	7.2

RIN: RNA integrity number
